# Successful control of juvenile dermatomyositis-associated macrophage activation syndrome and interstitial pneumonia: distinct kinetics of interleukin-6 and -18 levels

**DOI:** 10.1186/s12969-015-0048-2

**Published:** 2015-11-18

**Authors:** Hiroyuki Wakiguchi, Shunji Hasegawa, Reiji Hirano, Hidenobu Kaneyasu, Midori Wakabayashi-Takahara, Shouichi Ohga

**Affiliations:** Department of Pediatrics, Yamaguchi University Graduate School of Medicine, 1-1-1 Minamikogushi, Ube, Yamaguchi 755-8505 Japan; Division of Pediatrics, Yamaguchi-ken Saiseikai Shimonoseki General Hospital, Shimonoseki, Japan

**Keywords:** Cytokine, Juvenile dermatomyositis, Macrophage activation syndrome, Autoimmune-associated hemophagocytic lymphohistiocytosis, Interstitial pneumonia, IL-18, IL-6, KL-6

## Abstract

**Background:**

Macrophage activation syndrome (MAS) is the secondary hemophagocytic lymphohistiocytosis associated with rheumatic diseases. Recently, the different cytokine profiles between systemic juvenile idiopathic arthritis (sJIA)-associated MAS (sJIA-MAS) and juvenile systemic lupus erythematosus (JSLE)-associated MAS (JSLE-MAS) were reported. However, there is little information about juvenile dermatomyositis (JDM)-associated MAS (JDM-MAS).

**Case presentation:**

A 4-year-old girl with JDM was hospitalized because of fever, erythema, hepatosplenomegaly, cytopenia, liver dysfunction and coagulopathy. Bone marrow aspiration revealed appreciable numbers of activated and hemophagocytosing macrophages. She was diagnosed as having JDM–MAS complicated with interstitial pneumonia (IP) based on the findings of the elevation of serum Krebs von den Lungen-6 (KL-6) levels and chest computed tomography findings. We analyzed circulating levels of interleukin (IL)-2,4,6,10,18, tumor necrosis factor-α and interferon-γ in the patient. Hypercytokinemia occurred at the diagnosis of MAS and IP, showing with the prominent elevations of IL-6 and IL-18 levels. The cytokine profiles were distinct from those reported in patients with sJIA-MAS or JSLE-MAS. High-dose corticosteroid and cyclosporine therapy led to a drastic improvement of MAS with decreased IL-6 levels. Subsequent cyclophosphamide therapy successfully controlled IP, paralleled with the declining pattern of IL-18 and KL-6 levels.

**Conclusion:**

This is the first report to describe a successful treatment and the cytokine profile of JDM-MAS and IP. Serum IL-6 and IL-18 levels may be useful for predicting the disease activity of JDM-MAS and IP, respectively.

## Background

Macrophage activation syndrome (MAS) is the secondary hemophagocytic lymphohistiocytosis (HLH) associated with rheumatic diseases, which is a life-threatening complication of systemic inflammatory disorders [1]. It is clinically characterized by fever, hepatosplenomegaly, lymphadenopathy, cytopenia, intravascular coagulation and organ failure [1]. Juvenile dermatomyositis (JDM) is a multisystem disease of uncertain origin that results in chronic inflammation of the striated muscle and skin [2]. Although MAS has been reported to develop in various rheumatic diseases, it is most often complicated in the children with systemic juvenile idiopathic arthritis (sJIA) [1]. Juvenile systemic lupus erythematosus (JSLE) is also one of the underlying rheumatic diseases in which MAS occurs more frequently seen than in the others [3]. On the other hand, MAS is a quite rare complication in JDM patients [4].

Recently, the difference in the serum cytokine profiles between sJIA-associated MAS (sJIA-MAS) and JSLE-associated MAS (JSLE-MAS) was reported [5]. However, there have been no reports about the cytokine profiles in JDM-associated MAS (JDM-MAS) with interstitial pneumonia (IP). We present the first report about successful treatment and analysis of cytokine profile in a case with JDM complicated MAS and IP.

## Case presentation

### Case

**Fig. 1:**
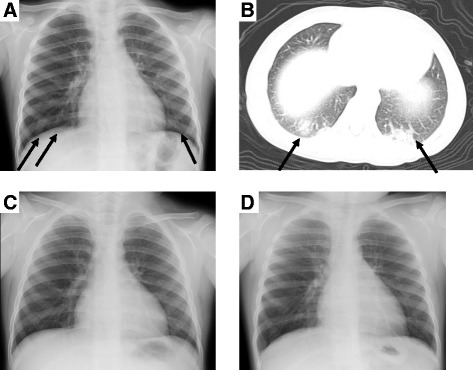
Chest radiograph (**a**) and computed tomography (**b**) show focal IP in the lower lobes during the acute phase, and no abnormalities at 4 months (**c**) and 8 months after the convalescent phase of JDM-MAS and IP (**d**). *Arrow* marks indicate focal IP

**Fig. 2:**
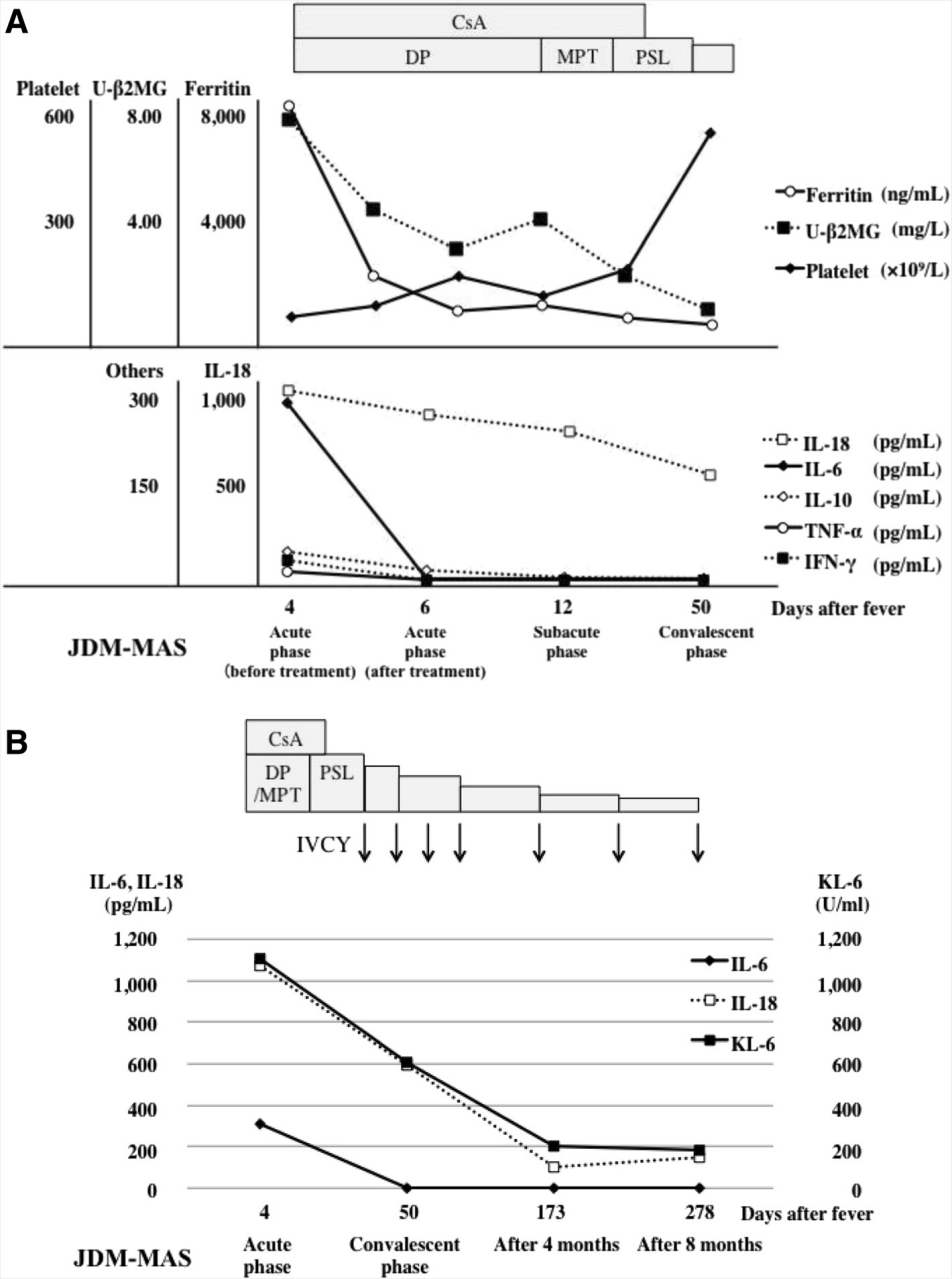
Sequential changes of the laboratory data and serum cytokine levels during the initial (**a**), and the overall treatment courses (**b**). IL-6 (♦), IL-18 (□) and KL-6 (■) were at higher levels at the onset of JDM-MAS and IP. Pearson correlation coefficient test indicated positive correlation of KL-6 with IL-18 (*r* = 0.997, *P* <0.01), but not IL-6 or the other cytokine levels. *CsA* cyclosporine A, *DP* dexamethasone palmitate, *MPT* methylprednisolone pulse therapy, *PSL* prednisolone, *IVCY* intravenous cyclophosphamide, *U-β*_*2*_ *MG* urinary β_2_-microglobulin, *IL* interleukin, *TNF-α* tumor necrosis factor-α, *IFN-γ* interferon-γ

A previously healthy 4-year-old girl exhibited butterfly like rash, heliotrope rash, Gottron’s papules, mild proximal muscle weakness, muscle grasping pain, and serum creatine kinase (CK) levels and erythrocyte sedimentation rate were elevated. This patient fulfilled the diagnostic criteria for dermatomyositis [6]. To fulfill the criteria, patients must meet more than 4 findings among 8 (1. proximal muscle weakness, 2. muscle grasping and spontaneous pain, 3. nondestructive arthritis or arthralgia, 4. elevated CK or aldolase level, 5. presence of systemic inflammatory signs (fever, elevated C-reactive protein, or elevated erythrocyte sedimentation rate), 6. myogenic changes on electromyogram, 7. anti Jo-1 antibody positive, 8. pathologic findings compatible with inflammatory myositis) with having shown the characteristic skin findings (heliotrope rash or Gottron’s papules). She was treated with high-dose oral prednisolone (PSL) followed by 2 courses of methylprednisolone pulse therapy (MPT). Subsequently, the skin rash and myositis gradually improved, and low-dose oral PSL was continued. Three months after MPT, she was emergently admitted to our hospital because of dyspnea, high fever, erythema, hepatosplenomegaly, cytopenia, liver dysfunction and coagulopathy. Hypoxemia, high Krebs von den Lungen-6 (KL-6) levels, and diagnostic imagings indicated progressive IP (Fig. 1a, b). Results of hematological examinations were as follows: leukocytes, 2.56 × 10^9^/L; hemoglobin, 13.4 g/dL; platelet count, 119 × 10^9^/L; D-dimer, 120.0 mg/L (reference range [rr]: 0–1 mg/L); alanine aminotransferase (ALT), 596 IU/L (rr: 5–43 IU/L); aspartate aminotransferase (AST), 1,154 U/L (rr: 12–34 IU/L); ferritin, 8,062 ng/mL (rr: 25–280 ng/mL); lactate dehydrogenase (LDH), 2,267 IU/L (rr: 115–217 IU/L); CK, 40 IU/L (rr: 41–123 IU/L); KL-6, 1,106 U/mL (rr: 0–499 U/dL). The urinary β_2_-microglobulin (U-β_2_MG) levels were elevated, 8.06 mg/L (rr: <0.29 mg/L). The tests for antinuclear antibody (1:80; speckle) and anti-melanoma differentiation-associated gene 5 (MDA5) antibody were positive. Anti-Jo-1 antibody and Epstein–Barr virus (EBV) DNA were negative. Bone marrow aspiration showed activated macrophages phagocytosing hematopoietic elements. She was diagnosed as having JDM-MAS, and progressive IP. Heparinization, dexamethasone palmitate (DP) and cyclosporine (CsA) therapy appreciably controlled the disease activity. Six days later, the flare-up of MAS such as decrease of platelet count and elevation of serum ferritin and U- β_2_MG levels required MPT for 3 consecutive days, and then successfully subsided (Fig. 2). The following intravenous cyclophosphamide (IVCY) therapy led to a clinical and radiological remission of IP (Fig. 1c, d).

We studied serum cytokine profiles in this patient during the acute (days 4, 6), subacute (day 12) or convalescent phase (day 50) of JDM-MAS with IP. Multi-target streaming protein quantitative technology (BD-Pharmingen Cytometric Bead Array; BD Biosciences, Franklin Lakes, NJ, USA) was used to detect the serum cytokine levels of interleukin (IL)-2,4,6,10, tumor necrosis factor-α (TNF- α), and interferon-γ (IFN- γ), following the manufacturer's instructions. The lower detection limits for IL-2, IL-4, IL-6, IL-10, TNF-α, and IFN-γ were 2.6, 2.6, 2.5, 2.8, 2.8, and 7.1 pg/mL, respectively. Serum levels of IL-18 were determined using enzyme-linked immunosorbent assay (ELISA) kits (Medical & Biological Laboratories, Co., Ltd., Nagoya, Aichi, Japan) according to the manufacturer's protocols. The lower detection limit was 12.5 pg/mL.

The clinical courses and the results of cytokine expressions in our patient with JDM-MAS and IP are shown in Fig. 2a, b. There was hypercytokinemia before the treatment and during the acute phase of JDM-MAS with IP. IL-6 and IL-18 were at prominent high levels compared with the elevated levels of IL-10, TNF-α and IFN- γ. IL-6 levels promptly decreased after the initial therapy. IL-18 levels sustained to be high after the start of effective treatments during the acute and subacute phases of JDM-MAS. On the other hand, the IL-18 levels decreased after IVCY therapy, in concert with the declining KL-6 levels and the clinical and radiographical improvement of IP (Fig. 2b).

## Discussion

There has been no report about the cytokine profile in JDM-MAS with IP. To our knowledge, only 5 patients with JDM-MAS were reported [4, 7–10]. There were 4 boys and 1 girl, the median age of whom was 12 years ranging from 7 to 14. MAS is a life-threatening condition, and the reported mortality rates reach 20 % [11]. On the other hand, all 6 patients including ours survived JDM-MAS after immunosuppressive therapy. The distinct changes between IL-6 and IL-18 levels during the treatment come of our patient may indicate the unique pathophysiology of JDM-driven complications.

Serum IL-18 concentrations in sJIA-MAS patients were reportedly higher than those seen in EBV-associated HLH (EBV-HLH) [12]. Monitoring the cytokine profile, including IL-18, may be useful for the differential diagnosis of sJIA-MAS from the other MAS/HLH. Although various proinflammatory cytokines are overexpressed in MAS patients, the prodominant elevations of TNF-α levels might be characteristic as shown in a patient with JSLE-MAS, compared to sJIA-MAS and EBV-HLH patients [5]. The cytokine profiles in MAS patients are considered to depend on the underlying rheumatic diseases. In the present case, both levels of IL-6 and IL-18 were prominently increased during the acute phase of JDM-MAS with IP before treatment. The serum levels of IL-18 may be associated with the disease activity of IP, but not JDM-MAS. Because Pearson correlation coefficient test indicated the positive correlation between serum KL-6 and IL-18 levels (*r* = 0.997, *P* <0.01) (Fig. 2b), we speculated that the leading cytokine may be IL-18 in sJIA-MAS, TNF-α in JSLE-MAS, and IL-6 in JDM-MAS, respectively. Serum IL-18 and TNF-α levels were dominantly elevated in patients with sJIA-MAS (average of 5 cases) and JSLE-MAS (one case), respectively (sJIA-MAS IL-18: >100,000, TNF-α: 0–10, IL-6: 10–50 pg/mL, JSLE-MAS, IL-18: 1,000–3,000, TNF-α: > 1,000, IL-6: 100–500 pg/mL) [5, 12]. IL-18 may be dominant cytokine in sJIA-MAS compared with JSLE-MAS and JDM-MAS. In our patient with JDM-MAS, the elevation of IL-18 and TNF-α levels was mild, compared with sJIA-MAS and JSLE-MAS. Serum IL-6 levels may reflect the disease activity of JDM-MAS. Further studies are needed to determine the distinct cytokine profile of MAS according to the underlying disease.

Our results suggest that IL-6 and IL-18 may play differential roles in the pathophysiology of JDM-MAS and IP, respectively. It has been reported that activated macrophages and monocytes produce proinflammatory cytokines, such as IL-6, TNF-α in MAS patients. On the other hand, IL-18 is produced not only from tissue-resident macrophages and bone marrow macrophages, but also from dendritic cells in the muscle, and lungs in patients with JDM [13]. In the present case, there was a positive correlation between serum IL-18 and KL-6 levels (Fig. 2b). In IP complicated with JDM, IL-18 is mainly produced from activated alveolar macrophages, and KL-6 is produced from type II pneumocytes and bronchial epithelial cells during regeneration of lung tissues [13]. The kinetics of these biomarkers may represent the tissues injury and regeneration occurring in the lungs of IP patients.

## Conclusion

This is the first report to describe the successful treatment and the cytokine profile in JDM-MAS with IP patient. Our results suggest that the elevations of serum IL-6 and IL-18 levels are associated with disease activity of JDM-MAS and IP, respectively. These results indicate that IL-6 and IL-18 may be useful for predicting treatment responsiveness in the pathophysiology of JDM-MAS with IP. However, the limitation of the present study is a single case analyzed. Therefore, the accumulation of further case reports is necessary for identifying the pathophysiology of JDM-MAS.

### Consent

Provided informed consent was obtained from the patient’s guardian for publication of this case report.

## Abbreviations

ALT: alanine aminotransferase
AST: aspartate aminotransferase
CsA: cyclosporine A
CK: creatine kinase
DP: dexamethasone palmitate
EBV: Epstein–Barr virus
ELISA: enzyme-linked immunosorbent assay
HLH: hemophagocytic lymphohistiocytosis
IFN-γ: interferon-γ
IL: interleukin
IP: interstitial pneumonia
IVCY: intravenous cyclophosphamide
JDM: juvenile dermatomyositis
JSLE: juvenile systemic lupus erythematosus
KL-6: Krebs von den Lungen-6
LDH: lactate dehydrogenase
MAS: macrophage activation syndrome
MDA5: melanoma differentiation-associated gene 5
MPT: methylprednisolone pulse therapy
PSL: prednisolone
rr: reference range
sJIA: systemic juvenile idiopathic arthritis
TNF-α: tumor necrosis factor-α
U-β_2_MG: urinary β_2_-microglobulin
